# A Single Bout of Fatiguing Aerobic Exercise Induces Similar Pronounced Immunological Responses in Both Sexes

**DOI:** 10.3389/fphys.2022.833580

**Published:** 2022-06-08

**Authors:** Lázaro Fernandes Lobo, Mariana Gomes de Morais, Lucas Soares Marcucci-Barbosa, Francisco de Assis Dias Martins-Junior, Luíza Martino Avelar, Erica Leandro Marciano Vieira, Felipe J. Aidar, Samuel Penna Wanner, Lucélia Scarabeli Silva, Maria Clara Noman, Bruno Muzzi Camargos, Kátia Michelle Freitas, William Antonio Gonçalves, Vanessa Pinho, Albená Nunes-Silva

**Affiliations:** ^1^ Laboratório de Inflamação e Imunologia do Exercício, Escola de Educação Física da Universidade Federal de Ouro Preto (EEFUFOP), Ouro Preto, Brazil; ^2^ Laboratório de Imunofarmacologia, Departamento de Bioquímica e Imunologia—Universidade Federal de Minas Gerais, Belo Horizonte, Brazil; ^3^ Programa de Residência Médica em Medicina de Família e Comunidade—Universidade Federal de Minas Gerais, Belo Horizonte, Brazil; ^4^ Laboratório Interdisciplinar de Investigação Médica, Faculdade de Medicina—Universidade Federal de Minas Gerais, Belo Horizonte, Brazil; ^5^ Departamento de Educação, Física da Universidade Federal de Sergipe, São Cristóvão, Brazil; ^6^ Laboratório de Fisiologia do Exercício, Escola de Educação Física, Fisioterapia e Terapia Ocupacional, Universidade Federal de Minas Gerais, Belo Horizonte, Brazil; ^7^ Faculdade de Medicina de Ribeirão Preto- USP, Center for Research in Inflammatory Diseases (CRID), Ribeirão Preto, Brazil

**Keywords:** immune response, myokines, skeletal muscle tissue, physical exercise (running), immunomodulation

## Abstract

**Introduction:** Physical exercise can acutely and chronically modulate immunological responses. Women and men have different innate and adaptive immune responses, and in this sense, these two groups may also have different acute immunological responses induced by exercise. In addition, it is essential to understand further whether the effects of physical exercise on the immune system responses depend on sex because limited scientific evidence on this topic is available. This information may allow athletes and coaches to improve the training process, mainly to understand if the physiological impact of given training stimuli in women is similar to that in men.

**Objective:** The present study aimed to investigate the acute effects of continuous submaximal exercise until fatigue on physiological and immunological parameters in amateur female and male runners.

**Methods:** This study included 18 female and 15 male volunteers. Each participant visited the laboratory on four consecutive days. The first visit consisted of medical history taking and explaining the study design. On the second visit, the participants were subjected to an incremental test to determine their maximal rate of oxygen consumption (VO_2max_) that was required to prescribe the intensity of the submaximal exercise protocol. On the third visit, the fatiguing exercise protocol was performed at 77%–80% of the VO_2max_. During this submaximal exercise, the heart rate, rating of perceived exertion (RPE), and blood lactate were recorded. Blood samples were collected before, immediately after, and 1 h after the fatiguing protocol to analyze the plasma levels of cytokines and creatine kinase (CK) and to count leukocytes. Finally, on the fourth visit, the participants underwent physical evaluations to measure their body composition using dual-energy X-ray absorptiometry (DXA) imaging.

**Results:** The average ages of the female and male groups were 34.2 ± 3.7 and 30.5 ± 4.3 years old, respectively. The female group ran 57 ± 27 min, while the male group ran 52 ± 15 min before fatiguing. In the female group, when comparing before and after the submaximal exercise, marked increases were observed in the following variables: heart rate (from 68.5 to 180.4 bpm), RPE (from 3.6 to 8.2), lactate (from 2.1 to 4.49 mmol/L), and CK (from 89.5 to 126.3 U/L). In addition, the female group showed an increased number of total leukocytes (from 7222.3 to 11162.9 × 10^6^/μl), neutrophils (from 4,403 to 6,480 × 10^6^/μl), and lymphocytes (from 2,342 ± to 3,562 × 10^6^/μl) from pre- to post-submaximal exercise. In the male group, similar elevations in psychophysiological variables were observed, as evidenced by comparing the heart rate (from 52.8 to 184.1 bpm), RPE (from 0.0 to 8.9), lactate (from 2.7 to 7.2 mmol/L), and CK (from 106.2 to 165 U/L) before and after the submaximal exercise. The male group also showed an augmented number of total leukocytes (from 6,245 to 8,050 × 10^6^/μl), neutrophils (from 3,335 to 4,128 × 10^6^/), and lymphocytes (from 2,191 to 3,212 × 10^6^/μl) when comparing pre- and post-submaximal exercise. There were no differences in the changes between women and men for these parameters.

**Conclusion:** The aerobically fatiguing exercise protocol induced pronounced changes in the heart rate, plasma levels of lactate and CK, total leukocyte count, especially the number of neutrophils and lymphocytes, in both sexes. The fatiguing exercise protocol also changed the plasma levels of IL-6 and IL-10 in the female and male groups. Under the present conditions, the physiological changes induced by fatiguing submaximal exercise, including the immunological changes, were not influenced by sex. This study shows that the same aerobic physical exercise can alter immunological parameters in women and men, and this response is similar between sexes.

## Graphical Abstract

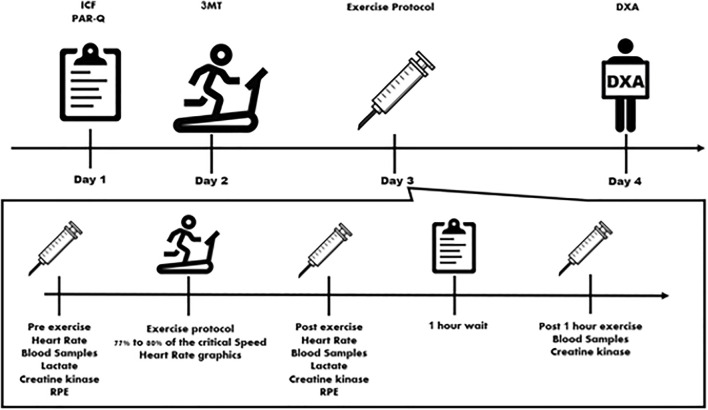

## 1 Introduction

Regular physical exercise improves the outcome of many of the emerging and increasingly prevalent clinical diseases. A massive body of recent findings demonstrates that one of the most important factors in this scenario is acute and chronic immunomodulation induced by exercise (Pedersen and Hoffman-Goetz, 2000). The immune system appears to be activated by physical stressors, and the immune/inflammatory response has become an essential component of physical training monitoring for athletes and recreationally active individuals. This low-grade inflammation status is associated with several types of obesity-related diseases such as diabetes, cardiovascular disease, cirrhosis, and cancer. Indeed, the literature suggests that control of this pathology-related inflammation can in part be ascribed by the release of immunogenic myokines (Bay and Pedersen, 2020).

Understanding the immune system is also crucial for promoting a healthy lifestyle, which includes adequate training, sufficient recovery, and a good nutrition**.** Plenty of evidence indicates that regular moderate-intensity physical exercise stimulates the immune system, culminating in protection against diseases, such as diabetes, hypertension, obesity, and many cancers (Pedersen and Febbraio, 2008; Pedersen and Febbraio, 2012). On the other hand, a sedentary lifestyle reduces life expectancy.

The modulation of the immune system by physical exercise, depends on important mediators, such as the cytokines, as well as the number of circulating leukocytes and their subpopulations, such as neutrophils, lymphocytes, monocytes, and eosinophils (Pedersen and Hoffman-Goetz, 2000; Brandt and Pedersen, 2010). These cytokines, which are produced and released by many cell types during and after physical exertion, mediate the beneficial effects of exercise on health. Cytokines can also influence metabolism and modify the production of cytokines in other tissues and organs, thus playing a fundamental role in regulating homeostasis and modulating the body's defense against chronic diseases (Gleeson et al., 2011; Benatti and Pedersen, 2015; Febbraio 2017). Many studies have shown increased levels of plasma concentrations of cytokines, such as interleukin-(IL)6, interleukin-(IL)10, tumor necrosis factor (TNF)α, and irisin after vigorous exercise (Peake et al., 2015; Blizzard LeBlanc et al., 2017). In addition, evidence from experimental studies in humans revealed elevated plasma concentration of several other cytokines, including IL-6, IL-8, IL-10, IL-15, CC-chemokine ligand (CCL)2, IL-1 receptor antagonist, calprotectin S100A9, and vascular endothelial growth factor (VEGF) (Hoffmann and Weigert, 2017). These changes have been observed in response to strength training (Fortunato et al., 2018; Marcucci-Barbosa et al., 2020) but also to endurance exercises that include both cycling (Gleeson, 2007; Zhao et al., 2012) and running (Kakanis et al., 2010; Marcucci-Barbosa et al., 2020). In general, systemic cytokine responses are more pronounced after exercises generating greater muscle damage, such as downhill running, eccentric exercise, and resistance training (Paulsen et al., 2012).

These findings have arisen from studies investigating men, likely because experimental studies in women are complex due to greater hormonal variations and specific responses caused by the menstrual cycle. As female physiology changes considerably during the month, some authors have claimed the need for studies on the particularities of physical exercise, sports performance, and immune system responses in women (Giraldo et al., 2008; Northoff et al., 2008; Giraldo et al., 2009). In this sense, the differences in immunological responses between men and women are probably influenced by biological factors (Katie and Flanagan, 2016). However, it is challenging to compare female and male data from the published studies, because few studies investigated the female population. Moreover, it is important to subject men and women to the same experimental design/exercise protocol to compare the immunological response between sexes.

Advanced knowledge of the biological sex differences in physiological responses will allow athletes and coaches to optimize the training process by understanding the physiological perturbations induced by training stimuli in women and men and how their body adapts/recovers after that. This knowledge will also help to understand possible sex-related differences in the ability of regular exercise to prevent disease occurrence. Given the limited scientific evidence comparing the exercise-induced immunological response between women and men, the present study investigated the acute effects of continuous submaximal exercise on physiological and immunological parameters in well-trained female and male runners. Herein, we hypothesized that the intense physical exercise could alter immunological markers in young adults and these alterations may show some differences between sexes.

## 2 Methods

### 2.1 Study Design

Each participant visited the laboratory four times, being one daily visit across consecutive days. The first visit consisted of history taking and explaining the study objectives and design. In addition, the participants had the opportunity to request clarifications about the research before signing the informed consent form and completing the Physical Activity Readiness Questionnaire (PAR-Q). On the second visit, the participants performed an incremental exercise test to determine the maximum rate of oxygen consumption (VO_2max_) that was required to prescribe the intensity of the fatiguing exercise. On the third visit day, the fatiguing exercise protocol at approximately 77%–80% of the VO_2max_ and the entire data collection were conducted. Finally, on the fourth visit, the participants underwent a physical evaluation to determine their body composition (i.e., percent body fat, body mass and height, and bone mineral density) using dual-energy X-ray absorptiometry (DXA) imaging.

### 2.2 Participants

Eighteen (18) women and fifteen (15) men took part in this study. The inclusion criterion for the female and male participants in this study was the ability to run 10 km in <50 min and <45 min, respectively, as indicated by their performance in a race taking place in the 6 months preceding the day of data collection. In addition, the volunteers could not have reported musculoskeletal injuries in the lower limbs and pelvis in the 6 months before the experiments. The participants agreed to the following recommendations for participation: do not consume alcohol; do not perform strenuous exercise; do not take anti-inflammatory or analgesic drugs; and do not consume either anabolic steroids or nutritional supplements.

The data was collected by a physician instructed to interrupt the tests if, according to guidance from the American College of Sports Medicine (ACSM, 2014), one of the following aspects has been observed: angina or angina-like symptoms; increased chest pain; an inability of the heart rate to increase with exercise; any physical or verbal manifestations of extreme fatigue; loss of movement quality; a request to stop the exercise; or test equipment failure. During this study, no volunteer presented angina-like symptoms, chest pain, or inappropriate heart rate responses. This study was approved by the Human Research Ethics Committee of the Federal University of Ouro Preto (UFOP), protocol number 1.881.170 (CAAE: 60064216.5.0000.5150). The volunteers signed an informed consent form, which stated they could quit participating in this project at any time. All experimental procedures were done in accordance with the “Guidelines and Regulatory Norms for Research Involving Human Beings” of the National Health Council (Resolution 466/2012).

The sample size calculation was performed a priori using data from pilot experiments (*n* = 5) investigating the effect of a fatiguing exercise session on the number of circulating leukocytes. The effect size (i.e., partial eta-squared; η_p_^2^) for time points—before, immediately after, and 1 h after the exercise—corresponded to 0.253. We then used the GPower software (v 3.1.9.7) to calculate the required sample size according to the following parameters: ANOVA (repeated measures, within-between interaction) with two groups (males vs. females) and the three time points mentioned above, alpha error = 0.05, power = 0.95, correlation between repeated measurements = 0.68; effect size = 0.253, and non-sphericity correction = 1. This calculation indicated that a total of 28 participants were needed.

### 2.3 Incremental Running Tests

An incremental exercise test was performed on a treadmill (Centurion 300 Micromed) to determine the participants’ cardiorespiratory capacity (VO_2max_). The protocol started with 3 min of preparatory activity at a speed of 4 km/h and an incline of 1%, and then the speed was increased to 6 km/h while the incline was kept constant at 1%. During the test, the speed was increased by 0.1% every 30 s until voluntary fatigue (Castagna et al., 2009). Gas analysis was performed using an ergospirometer (Metalyzer 3B Cortex). VO_2max_, respiratory exchange rate, heart rate, electrocardiogram, blood pressure, and perceived exertion were measured during this fatiguing exercise protocol. A medical cardiologist carried out the test at a sports medicine clinic accompanied by a researcher who conducted a brief interview.

#### 2.3.1 Fatiguing Exercise Protocol

On the third visit, the participants arrived at the lab after having their habitual breakfast. After a 5-minute warm-up at <5 km/h, the speed at which the participants should exercise was adjusted on the treadmill dashboard. The treadmill automatically increased the speed until reaching the individual prescribed speed. The participants performed the constant exercise at an intensity corresponding to 77%–80% of VO_2max_ until they had voluntarily fatigued (e.g., inability to maintain the predetermined speed or a score of 10 on the perceived exertion scale) or asked to stop exercising (Miranda-Castro et al., 2022). Throughout the exercise protocol, the volunteers could drink water *ad libitum* but did not have access to information such as speed, running time, and heart rate.

### 2.4 Heart Rate and Rating of Perceived Exertion

The participant’s heart rate was monitored throughout the fatiguing exercise protocol using a chest strap heart rate monitor (Polar m600 or V800). FlowSync version 3.0 (Polar^®^) was used to generate graphs of the heart rate values attained during the submaximal exercise. The maximum heart rate values were derived from the following equation [208 - (0.7 × age)]. The RPE was obtained on a 10-point Borg’ scale (CR-10, Borg, 1998). During the 1 h that followed the submaximal exercise protocol, the volunteers did not perform any physical activity. RPE was collected only before and immediately after the voluntary fatigue. This scale measures the perceived effort related to the exercise and has been widely used as a marker of intensity (Borg, 1998).

### 2.5 Blood Sample Collection

A qualified health professional collected, transported, and stored the blood samples. Before exercise, immediately after, and again 1 h after the fatiguing exercise protocol, the participants' peripheral venous blood was collected through venipuncture in alternate arms using two vacutainer tubes (5 ml each) containing heparin as an anticoagulant. One of the tubes (fresh blood) was used for counting white blood cells and their subpopulations, while the other tube was centrifuged and stored for later analysis of CK and cytokines. The hematocrit and hemoglobin data from the blood count were used to correct the blood parameters by changes in the plasma volume, such as the plasma levels of creatine kinase and cytokines.

### 2.6 Lactate Concentration Analyses

Fingertip capillary blood samples were collected using a lancet to pierce the finger. The lactate concentrations in these samples were measured by reflectance photometry before and immediately after exercise, using reagent test strips inserted into a portable lactometer (*AccutrendPlus, Roche Diagnostic*). This measurement was performed within a maximum of 1 min after capillary blood collection.

### 2.7 Creatine Kinase Analysis

Plasma sample that was stored in a 1.5 ml *Eppendorf* in a −80°C freezer was used for CK analysis. The CK levels were measured at the Pilot Laboratory of Clinical Analysis (LAPAC) at the School of Pharmacy of the Federal University of Ouro Preto (UFOP). This analysis was conducted using a CK liquid reagent on an automated high-throughput chemistry analyzer (COBAS Integra 400-plus, Roche Diagnostics, United States).

### 2.8 Cytometric Bead Array Analysis for Measuring Cytokines

Plasma sample that was stored in a 1.5 ml *Eppendorf* in a −80°C freezer was used for cytokines analysis. The levels of Il-2, IL-4, IL-6, IL-10, TNF-α, and IFN-γ were measured using a *BD* ™*Cytometric Bead Array Kit* (CBA, BD Biosciences, San Diego, United States). The samples were diluted 1:5 in the test diluent. In parallel, a series of nine standard dilutions were prepared to obtain the standard curve. Mixed beads of cytokine catches were added, and the samples were incubated at 25°C in the dark for 90 min. The samples were then washed with a washing buffer and centrifuged for 7 min at 600 g at 18°C. The supernatant was discarded, and the beads were incubated with eight anti-cytokine antibodies conjugated with phycoerythrin at 25°C for another 90 min in the dark. The beads were then resuspended in wash buffer and immediately analyzed on a FACScan ™ flow cytometer. These measurements were performed in the Interdisciplinary Laboratory for Medical Research at the Faculty of Medicine of the Federal University of Minas Gerais.

### 2.9 Full Blood Count Analysis

The counting of the immune cells and their subpopulations was performed in a fresh blood sample on the same day of the submaximal exercise protocol. The analysis was carried out by a commercial laboratory hired to conduct this examination. The number of white cells and their subpopulations, as well as the hematocrit and hemoglobin concentration, were measured (Fortunato et al., 2018).

### 2.10 Dual-Energy X-Ray Absorptiometry Imaging

The assessment of body composition using DXA imaging is increasingly common in the scientific community. This method allows accurate measurements of total body lean mass, lower limb region muscle mass, and the relative lean mass index, with high correlations reported with computed tomography and image resonance and lower radiation emission compared to computed tomography (El Maghraoui and Roux, 2008). In this study, DXA was performed by a qualified and experienced professional using a GE/Healthcare Model iDXA Densitometer (serial number ME + 210584) in a private hospital in the city of Belo Horizonte (Minas Gerais, Brazil). Body mass, height, and composition (bone mineral density, muscle mass, and percent body fat) were measured.

### 2.11 Statistical Analysis

GraphPad Prism version 6.0 was used to test the normality of the data. D’Agostino & Pearson tests were applied with *α* = 0.01. Wilcoxon tests were used for statistical analysis. Data with more than two time points that showed a normal distribution were analyzed by two-way mixed-design analysis of variance (ANOVA), with repeated measures used to compare intragroup data. The results were expressed as means ± standard deviations, with *p* values < 0.05 considered statistically significant.

## 3 Results

### 3.1 General Participant Characteristics and Physiological Data of the Participants

Table 1 shows the average and standard deviation values of general characteristics and physiological data for women and men participants. In the male group, the average age was 30.5 ± 4.3 years, weight was 71.6 ± 5.9 kg, percent body fat was 18.1 ± 4.5%, total muscle mass was 55.6 ± 4.1 kg, whereas lower limb muscle mass was 19.4 ± 1.8 kg. These values in the female group were 34.2 ± 3.7 years, 57.8 ± 6.2 kg, 27.3 ± 4.8%, 40.0 ± 4.1 kg, and 14.1 ± 1.6 kg, respectively. The VO_2max_ was also recorded during the incremental exercise test and corresponded to 54.2 ± 5.5 and 38.2 ± 3.6 mLO_2_·kg^−1^·min^−1^ in the male and female groups, respectively (Table 1).

**TABLE 1:** Characterization of the female and male groups. The absolute values and means of each characteristic are shown.

	Male				Female				
	Minimum	Mean	Maximum	S.D.	Minimum	Mean	Maximum	S.D.	*p* =
Age (years)	23.0	30.5	39.0	±4.3	25.0	34.2	40.0	±3.9	0.018^∗^
Body mass (kg)	61.3	71.6	82.5	±6.7	49.1	57.8	72.2	±6.4	0.0001^∗^
Muscle mass (%)	49.2	55.6	63.9	±4.1	61.4	69.6	77.0	±4.6	0.0001^∗^
Body fat (%)	9.4	18.1	25.3	±4.5	19.5	27.3	35.7	±5.0	0.0001^∗^
Lower limb muscle mass (kg)	17.2	19.4	22.8	±1.8	11.7	14.2	18.7	±1.9	0.0001^∗^
Resting heart rate (bpm)	41.0	53.0	61.0	±5.0	56.0	68.5	93.0	±10.1	0.0001^∗^
VO_2_ (ml·kg·min)	48.9	54.2	71.3	±5.5	31.0	38.2	44.0	±3.6	0.0001^∗^

*^∗^p <* 0.05 significantly different comparing female and male groups.

### 3.2 Ovarian Hormone Levels

**FIGURE 1:**
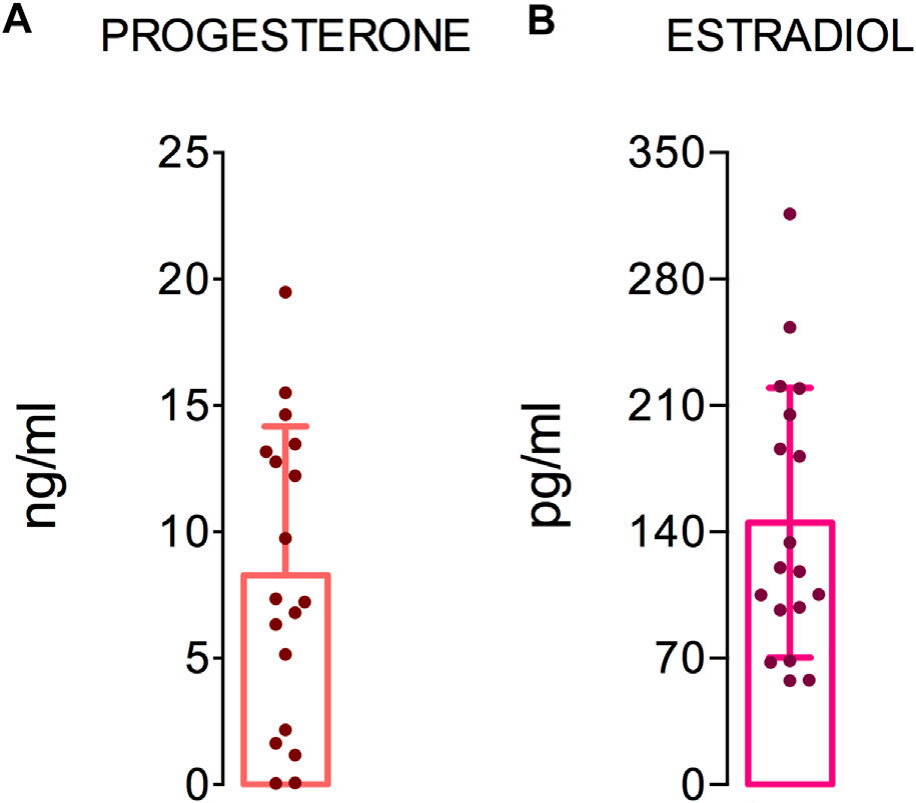
Ovarian hormones levels. The graphs show the hormone levels in 18 women. The dashed (red) and dotted (black) lines show the reference values of progesterone and estradiol for the luteal and follicular phase of the menstrual cycle, respectively. The median, minimum, maximum, and first and third quartile values are shown.

Women were tested in the same phase of the menstrual cycle to ensure that the fluctuations in hormone levels did not interfere with the immunological responses investigated. Therefore, we evaluated the ovarian hormones in the female group (Figure 1). The mean plasma levels of progesterone and estradiol were 8.2 ± 5.7 ng/ml (Figure 1A) and 145.1 ± 72.6 pg/ml (Figure 1B), respectively. According to these data, the women were in the luteal phase of their menstrual cycle.

### 3.3 Fatiguing Aerobic Exercise-Induced Physiological Changes

**FIGURE 2:**
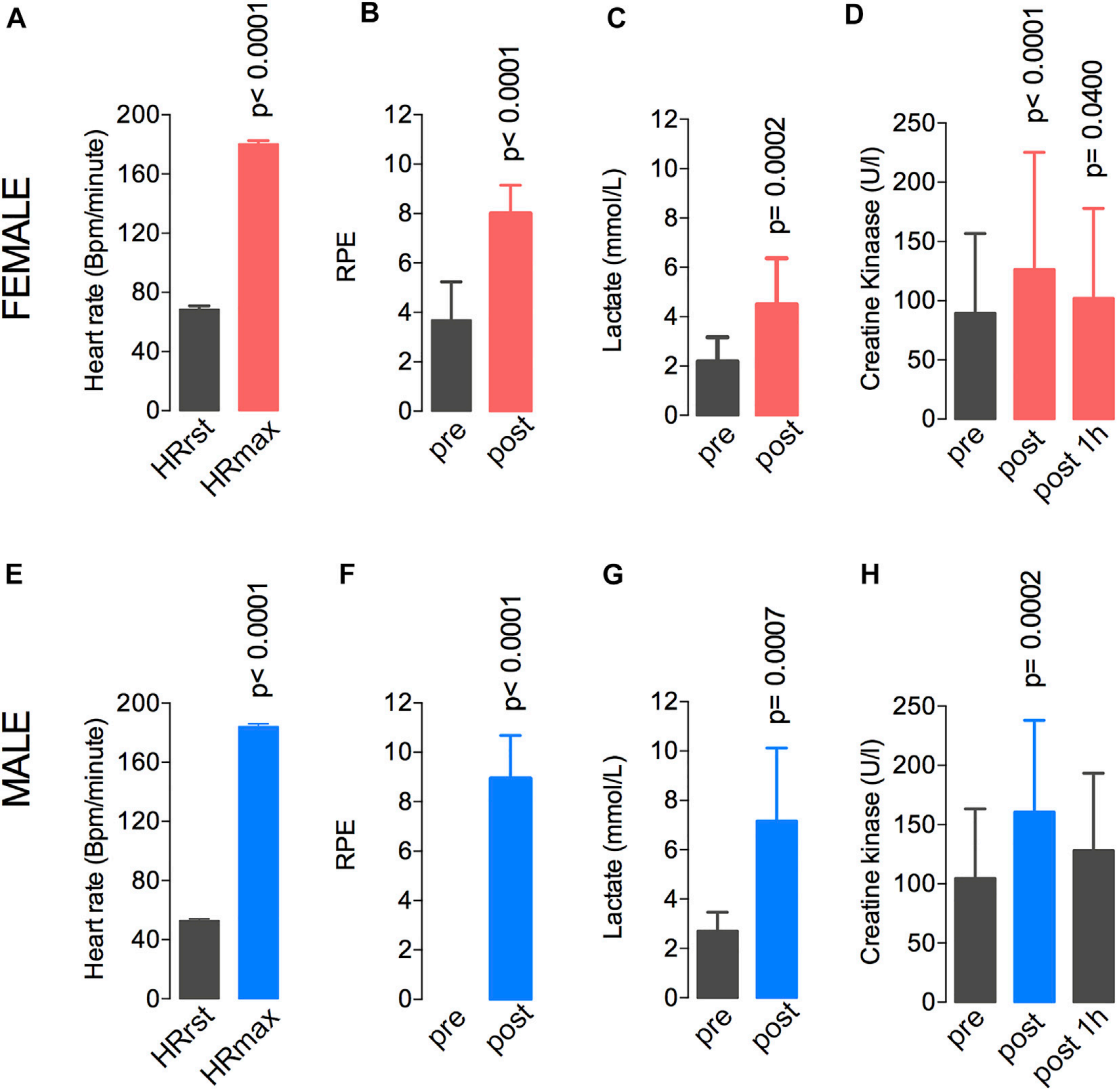
A single session at 77%–80% of the VO_2max_ resulted in elevated heart rate **(A,E)**, RPE **(B,F)**, lactate levels **(C,G)**, and creatine kinase levels **(D,H)** in both male and female groups. Data are shown as means and standard deviations, with *p* < 0.05 indicating statistical significance. ^∗^Difference between time-points. RPE, rate of perceived exertion.

The heart rate, the rate of perceived exertion (RPE), and the plasma levels of lactate and creatine kinase were evaluated during the fatiguing exercise on the treadmill in both groups (Figure 2). In the female group, between the pre-test and post-test, the heart rate increased from 68.5 to 180.4 bpm (Figure 2A), the RPE increased from 3.6 ± 1.5 to 8.2 ± 1.1 (Figure 2B), the lactate level increased from 2.1 ± 0.9 to 4.4 ± 1.8 mmol/L (Figure 2C), and the CK levels increased from 65.7 ± 67.1 to 96.9 ± 98.9 U/L (Figure 2D). In the male group, the heart rate increased from 52.8 to 184.1 bpm (Figure 2E). The RPE increased from 0.0 ± 0.0 to 8.9 ± 1.8 (Figure 2F), the lactate level increased from 2.7 ± 0.8 to 7.2 ± 2.9 mmol/L (Figure 2G), and the CK levels increased from 106.2 ± 60.6 to 165 ± 78.3 U/L (Figure 2H). Also, the expected exercise-induced changes in the heart rate variability (HRV) data were observed in the male participants. These changes included reduced parasympathetic-related indices (i.e., RMSSD and HF band; Shaffer and Ginsberg, 2017) and lower total power after the fatiguing exercise (Supplementary Table S1).

### 3.4 Effects of Fatiguing Aerobic Exercise on the Number of Leukocytes and Their Subpopulations

**FIGURE 3:**
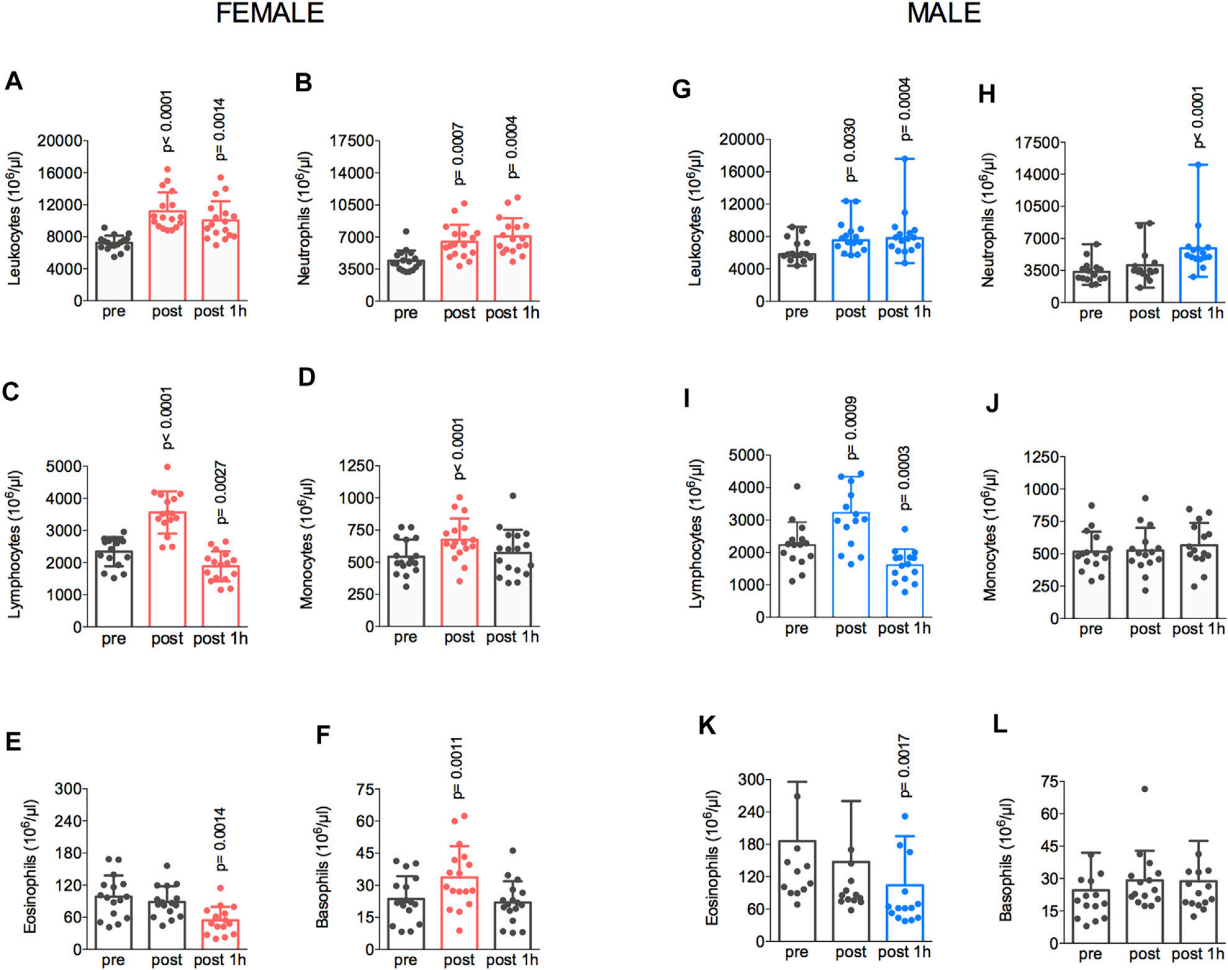
The exercise protocol resulted in elevated total leukocyte **(A,G)** and lymphocytes **(C,I)** count immediately after the session. Neutrophil **(B,H)** count increased at 1 h post-test in both male and female groups. Monocytes **(D,J)** and basophils **(F,L)** count also increased 1-h post-test in the female group. Eosinophils **(E,K)** reduced at 1 h post-test in both male and female groups. Data are shown as means and standard deviations, with *p* < 0.05 indicating statistical significance. ^∗^Difference between time-points.

The fatiguing exercise protocol at 77%–80% of the VO_2max_ changed the count of circulating immune cells in both groups. The changes in cell counts from the pre-exercise to post-exercise, from the post-exercise to 1 h after, and from the pre-exercise to 1 h after are shown in Figure 3. In the female group, an increase in the total leukocyte count was also observed between pre- and post-exercise (7,222 ± 910 vs. 11,162 ± 2,374 × 10^6^/μl) and the cell number remained elevated at 1 h after the exercise (10,040 ± 2,395 × 10^6^/μl) (Figure 3A). Increased counts of neutrophils were also observed at post-exercise (4,403 ± 1,122 vs. 6,480 ± 1,860 × 10^6^/μl) (Figure 3B). The number of lymphocytes also increased (2,342 ± 456 vs. 3,562 ± 655 × 10^6^/μl) after the treadmill running and then decreased significantly (1,887 ± 465 × 10^6^/μl) (Figure 3C) at 1 h after the exercise compared to the pre-exercise value. The monocytes count also increased when comparing pre- and post-exercise (542 ± 134 vs. 674 ± 165 × 10^6^/μl) (Figure 3D). The eosinophil count did not change from pre- to post-exercise (98 ± 39 vs. 88 ± 29 × 10^6^/μl), but it reduced (54 ± 25 × 10^6^/μl) 1 h after the treadmill running (Figure 3E). Finally, the basophils count also increased from 23 ± 10 to 33 ± 14 × 10^6^/μl at post-exercise relative to pre-exercise values (Figure 3F). In the male group, compared to the pre-exercise, the post-exercise total leukocyte count was increased from 6,245 ± 1,454 to 8,050 ± 2,170 × 10^6^/μl, and it remained elevated at 1 h after the exercise (8,191 ± 3,106 × 10^6^/μl) (Figure 3G). The neutrophil count also increased from 3,335 ± 1,245 to 4,128 ± 2,031 × 10^6^/μl (Figure 3H), whereas the lymphocyte counts also increased from pre- to post-exercise (from 2,191 ± 716 to 3,212 ± 1,153 × 10^6^/μl), and then it reduced significantly (1,579 ± 496 × 10^6^/μl) at 1 h after the test, compared to the pre-exercise value (Figure 3I). No changes were observed for the number of monocytes (504 ± 154 vs. 652 ± 165 × 10^6^/μl) (Figure 3J). The eosinophil count decreased from 189 ± 113 to 107 ± 93 × 10^6^/μl 1 h after relative to pre-exercise (Figure 3K). Finally, no changes were observed for basophil counts (22 ± 10 vs. 29 ± 14 × 10^6^/μl) (Figure 3L).

### 3.5 Effects of Fatiguing Aerobic Exercise on Plasma Cytokine Levels

**FIGURE 4:**
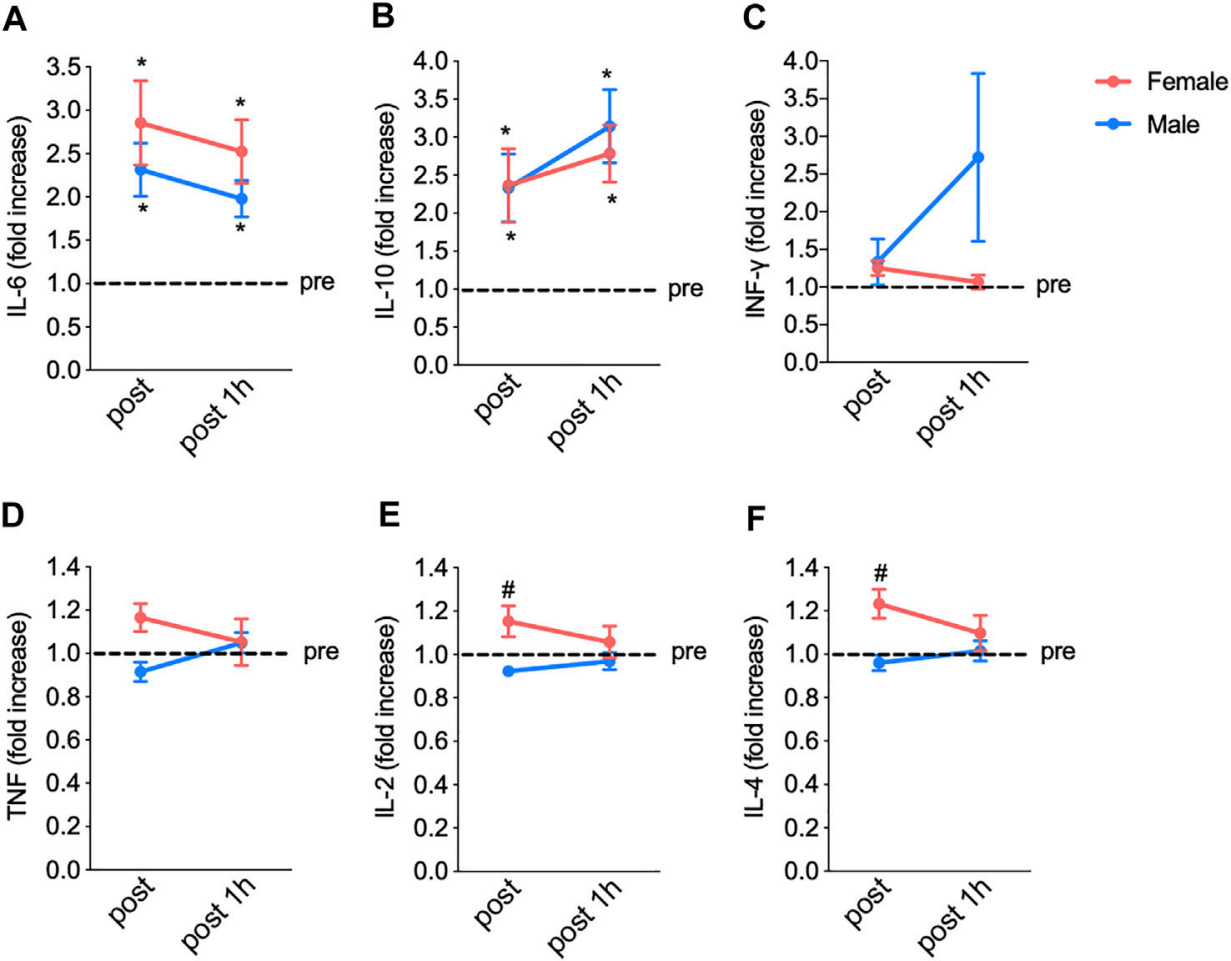
The fatiguing exercise protocol results in increased interleukin IL-6 **(A)** and IL-10 **(B)** levels immediately after the exercise compared to pre-test values in both groups. No significant changes in INFγ **(C)**, TNFα **(D)**, IL-2 **(E)**, and IL-4 **(F)** concentrations were observed. Data are shown as means and standard deviations, with *p* < 0.05 indicating statistical significance. IFN, interferon; TNF, tumor necrosis factor; IL, interleukin.

These results reinforced that running on a treadmill indeed modulates immunological responses, as further evidenced by the changes in the plasma levels of cytokines in both women and men (Figure 4). In the female group, the fatiguing exercise protocol increased the levels of IL-6 (from 1.0 to 2.8-fold, from pre-exercise to post-exercise) (Figure 4A) and IL-10 (from 1.0 to 2.3-fold) (Figure 4B); these levels remained elevated (2.5- and 2.8-fold, respectively) at 1 h after the exercise. In addition, no exercise-induced changes were observed in the plasma levels of IFN-γ, (Figure 4C), TNF-α (Figure 4D), IL-2 (Figure 4E), and IL-4 (Figure 4F). In the male group, the fatiguing exercise increased the levels of IL-6 (from 1.0 to 2.3-fold) (Figure 4A) and IL-10 (from 1.0 to 2.3); these levels remained elevated (2.0- and 2.1-fold, respectively) at 1 h after the exercise. No exercise-induced changes were observed in the levels of IFN-γ (Figure 4C), TNFα (Figure 4D), IL-2 (Figure 4E), and IL-4 (Figure 4F). A two-way ANOVA analysis also showed the differences between female and male groups in the IL-2 and IL-4 levels when comparing pre-test and post-test.

### 3.6 Effects of Fatiguing Aerobic Exercise on IFN-γ, TNFα, IL-6, IL-2, IL-4, and IL-10 Levels

**FIGURE 5:**
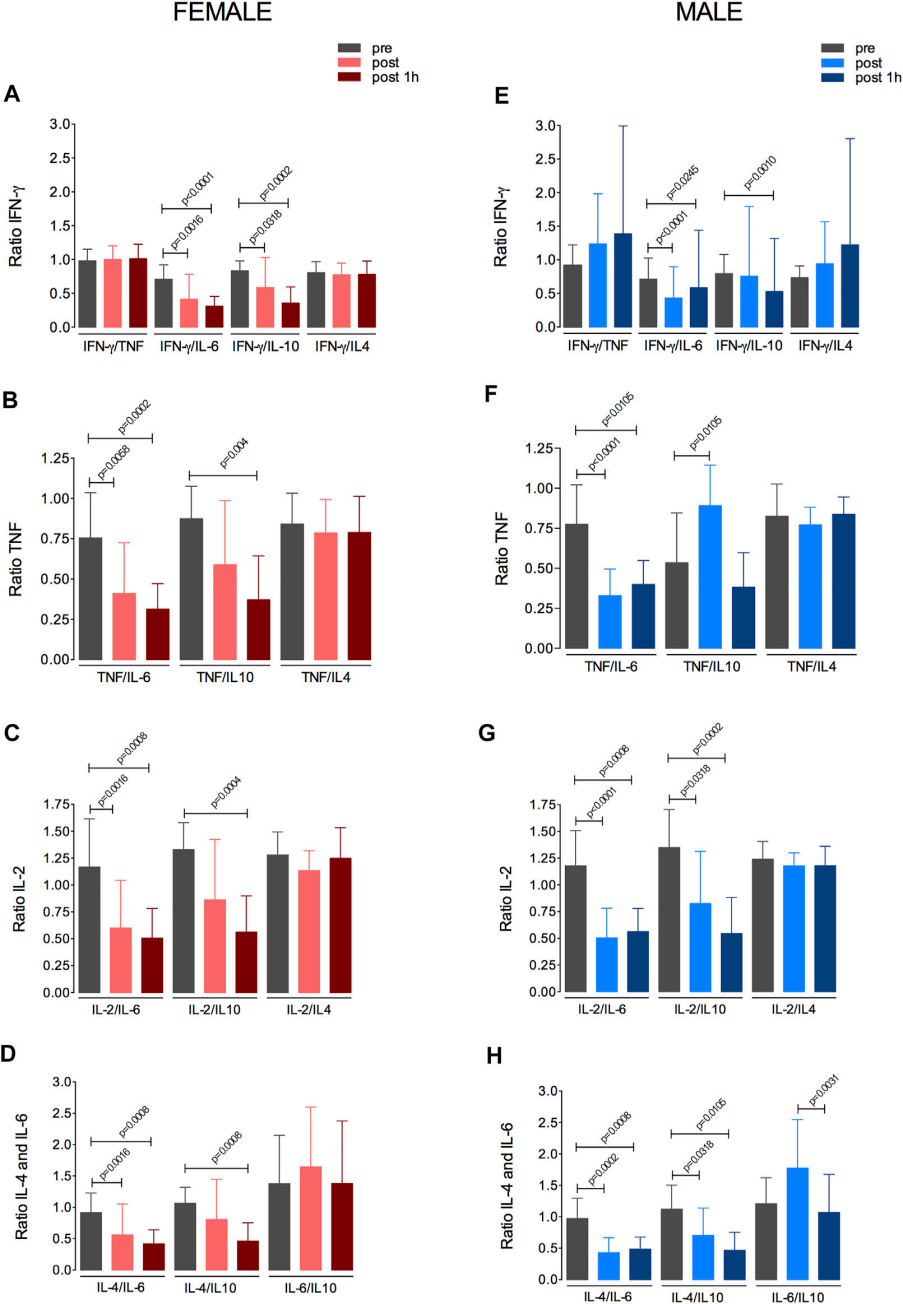
Changes in IFNγ, TNFα, IL-6, IL-2, IL-4, and IL-10 levels in response to the fatiguing exercise protocol. Changes in the levels of IFNγ **(A,B)**, TNFα **(C,D)**, IL-2 **(E,F)**, IL-4 **(G,H)** in the female and male groups are shown. IFN, interferon; TNF, tumor necrosis factor; IL, interleukin.

The possible relationships between the changes in IFN-γ, TNFα, IL-6, IL-2, IL-4, and IL-10 levels in response to the fatiguing exercise were investigated in both sex groups (Figure 5). The changes in the levels of these cytokines following the fatiguing aerobic exercise were similar between women and men. In addition, in both groups, IL-6 appeared to modulate the cytokine response, as IL-6 levels were directly related to those of IFN-γ (Figures 5A,B), TNFα (Figures 5C,D), IL-2 (Figures 5E,F), and IL-4 (Figures 5G,H).

## 4 Discussion

Most publications investigating the relationship between physical exercise and immune response involve male participants, likely because experimental studies in women are more complex due to large hormonal fluctuations and specific responses during the menstrual cycle. In this sense, few studies have evaluated the effect of a single bout of aerobically fatiguing exercises on the immunological response in both sexes. The main results of the present study were: 1) the aerobic exercise induced pronounced changes in leukocyte counts in both sexes; 2) the aerobic exercise also induced important changes in plasma cytokine levels in both sexes; 3) the changes and their magnitudes were similar between the sexes; and finally, 4) IL-6 cytokine appears to play a regulatory role in the exercise-induced immune response in women and men.

The present study focused on comparing the immunological response of women and men after fatiguing treadmill running, and this is an emerging topic in the field of exercise immunology. The current findings may help understand possible immunological differences between female and male athletes and physically active individuals after a physical exercise session. Of note, the immune response is involved in several training-induced adaptations, including those occurring in skeletal muscles (Chazaud, 2020). Moreover, our findings are also crucial to understand whether biological sex modulates the role played by exercise in preventing or treating diseases associated with augmented systemic inflammation, such as obesity and type 2 diabetes (Pedersen, 2012).

As expected, a single session of aerobic exercise until fatigue produced marked changes in psychophysiological parameters. From pre- to post-exercise, we observed increased heart rate, RPE, plasma lactate levels, and circulating CK levels. All these responses agree with the findings of previous studies in which the individuals were subjected to a single session of vigorous exercise (Fortunato et al., 2018; Marcucci-Barbosa et al., 2020). In fact, the RPE and heart rate values at fatigue above 8 and 180 bpm, respectively, confirm that the women and men participants were exercising at their maximum or near-maximum effort when they stopped running. Interestingly, CK levels were already augmented immediately after the exercise, even though evidence suggests that this muscle damage peaks around 24 h after exercise on a treadmill (Hackney et al., 2019; Aloulou et al., 2020) or a soccer match-play (Silva et al., 2018). However, data are scarce on the differences in exercise‐induced acute immune response between female and male groups.

Vigorous physical exercise modulates some fundamental aspects of the immune system, such as the number of circulating white blood cells and plasma levels of cytokines (Kakanis et al., 2010). However, data on sex differences in exercise-induced acute immune responses are scarce. Because women and men have different immunological responses to foreign and self-antigens and show distinctive innate and adaptive immune responses (Klein and Flanagan, 2016), we expected that they also should respond differently to a physical exercise session.

The present results showed that the fatiguing aerobic exercise induced pronounced changes in the number of white blood cells (Figure 3), but these changes were similar between sexes. Our findings agree with the observation that 90-min moderate-intensity cycling induced similar increases in immune cell counts between men and women not using contraceptives, except for a 38% greater lymphocyte response in these women. However, neutrophil, monocyte, and lymphocyte responses to exercise during the luteal phase in women using contraceptives were greater than those in men (Timmons et al., 2005). Moreover, our data also disagree with findings obtained in children and adolescents (Timmons et al., 2006). Exercise consisting of cycling for 60 min at 70%VO_2max_ increased the number of lymphocytes and CD3j, CD16^+^, and CD56^+^, with greater increases reported in adolescent girls than boys, but no differences between younger girls and boys. In addition, the exercise-induced increases in the counts of total leukocyte, lymphocyte, CD3j, CD16^+^, and CD56^+^ were at least 35% greater in girls than in boys with similar pubertal status (Timmons et al., 2006). Probably the difference between the results in these studies could be associated with the intensity of the exercise, once our volunteers exercised until fatigue.

Interestingly, our findings are in accordance with those provided by Gleeson et al. (2011). In the latter study, eighty physically active individuals (46 men, 34 women) trained on average 10 h/week at moderate-to-vigorous intensities; after that, differential leukocyte counts and lymphocyte subsets were determined. While the total blood leukocyte, neutrophil, monocyte, and lymphocyte counts did not differ between sexes, men had more B and NK cells. Consistent with the present results, the authors concluded that most aspects of immunity were similar between the sexes in an athletic population, with some differences in a few immune variables (Gleeson et al., 2011).

Finally, Abbasi et al. (2016) showed that running a half-marathon significantly increased total leukocyte count for 3 h post-exercise in both male and female athletes, with no sex-specific differences in the number of any immune cell population or total leukocytes. Neutrophil numbers and percentages were both significantly increased at 30 min and 3 h post-exercise, while the percentages of monocytes and lymphocytes were decreased at the same time points. The lymphocyte percentages returned to the pre-exercise levels at 24 h post-exercise. Once again, these (sem virgula entre once e again) results did not show a difference between sexes.

Our findings are in line with the similar exercise-induced increase in plasma IL-6 between men and women not using contraceptives and with IL-6 comparisons made between sexes in a pediatric population (Timmons et al., 2005). Our analyses indicate that most cytokines responded similarly in women and men, except for IL-2 and IL-4. IL-2 is a cytokine signaling molecule that regulates the activities of leukocytes, often lymphocytes. IL-2 is part of the body’s natural response to microbial infection, and in discriminating between foreign (“non-self”) and “self.” The major sources of IL-2 are activated CD4^+^ T cells and activated CD8^+^ T cells. IL-4 is a cytokine that functions as a potent regulator of immunity secreted primarily by mast cells, Th2 cells, eosinophils, and basophils. IL-4 is an important player in leukocyte survival under both physiological and pathological conditions, such as Th2 cell-mediated immunity, IgE class switching in B cells, and tissue repair and homeostasis through “alternative” macrophage activation. IL-4 is produced primarily by mast cells, T_h_2 cells, eosinophils, and basophils (Liu et al., 2021).

It is important to mention here that the main aim of this study was to compare the immune response between female and male groups. The current statistical analyzes clearly show that the immune response between the female and male groups is similar. Only the IL-4 and IL-2 responded differently between groups. We reinforce that we tried to ensure that the female group was in the same phase of the menstrual cycle to avoid possible effects on the immune response. The novelty of this study is to clearly show that the same aerobic physical exercise protocol can alter immunological parameters in women and men and this response is similar between sexes.

The strongest aspect of this study is that the same physical exercise was used in male and female volunteers. Another important aspect is that the female group was in the same phase of the menstrual cycle. On the other hand, one important limitation of the currently study is the fact that we analyzed only the luteal phase of the menstrual cycle. From a practical perspective, these results suggest that the training process of immune system could be similar in both groups. As future perspectives, it is important to better understand the chronic application of training process on immune system and compare the responses between female and male population.

## 5 Conclusion

The aerobically fatiguing exercise protocol induced pronounced changes in the immune parameters—such as the total leukocyte count, especially the neutrophil and lymphocyte numbers—and in the plasmatic cytokine levels of both female and male groups. The results also show that these responses were similar between both sexes.

## Data Availability

The original contributions presented in the study are included in the article/Supplementary Material, further inquiries can be directed to the corresponding author.
