# Supplementary material for: A Single Bout of Fatiguing Aerobic Exercise Induces Similar Pronounced Immunological Responses in Both Sexes
Source: Front Physiol. 2022 Jun 8;13:833580. doi: 10.3389/fphys.2022.833580 (PMC9213785; doi:10.3389/fphys.2022.833580)
Supplement: Supplementary file 1 [file Table1.pdf]

This is supplemental material for the manuscript entitled "A single bout of fatiguing aerobic exercise induces similar pronounced immunological responses in both sexes".

The heart rate variability (HRV) was measured in nine male participants before and after the fatiguing exercise (Supplementary Table 1). For these measurements, the participants remained seated for 5 minutes, with a normal breathing rate, in silence, and without body movements.

To collect the heart rate data, a chest strap (Polar® H7, Kempele, Finland) connected to a recording watch (Polar® V800) was used to record R–R intervals continuously. These data were transferred to software (Polar® ProTrainer) and exported for subsequent analysis using the Kubios HRV (version 2.0) developed by the Biosignal Analysis and Medical Imaging Group at the Department of Applied Physics, University of Kuopio, Finland.

The data were visually inspected to identify ectopic beats and artifacts (which did not exceed 2% of the recorded data), and those identified were removed and replaced by interpolation of their respective adjacent R–R intervals. The HRV analysis in the time domain consisted of determining the average R–R intervals (mean RR), the standard deviation of all normal-to-normal (NN) intervals (SDNN), and the square root of the mean of the sum of the squares of differences (RMSSD) between adjacent NN intervals. A fast Fourier transform of the RR signals was used for analyzing HRV in the frequency domain. The spectral response provided by the analysis was divided into three bands: very low frequency (VLF; 0.00–0.04 Hz), LF (0.04–0.15 Hz), and HF (0.15–0.40 Hz). Next, the total power (i.e., VLF + LF + HF) and the LF-to-HF ratio were calculated, as well the normalized LF and HF. For example, the normalized LF (%) was calculated as follows: LF n.u. = $[LF / (LF + HF)] \times 100$.

Supplementary table 1. The heart rate variability parameters in male participants (n = 9) before and after the fatiguing exercise.

Parameter	Pre-exercise	Post-exercise	*P*-value
Mean RR (ms)	932.4 ± 64.9	656.9 ± 65.5 *	0.003
SDNN (ms)	74.2 ± 10.1	40.5 ± 6.1 *	0.009
RMSSD (ms)	61.4 ± 11.5	26.2 ± 5.2	0.005
VLF (ms^2)	1,003 ± 493	83 ± 28	0.108
LF (ms^2)	4,329 ± 1,010	1,599 ± 466	0.060
HF (ms^2)	1,154 ± 360	262 ± 62 *	0.033
Total power (ms^2)	6,486 ± 1,730	1,944 ± 524 *	0.046
LF-to-HF ratio	4.64 ± 0.85	6.11 ± 0.90	0.290
LF (n.u.; %)	79.1 ± 3.7	84.4 ± 2.1	0.279
HF (n.u; %)	20.9 ± 3.7	15.6 ± 2.1	0.286

Values are expressed as means ± SE. Comparisons were made using paired Student's t-tests, and the * means a significant difference from pre-exercise values ($P < 0.05$). Legend: HF = high frequency, LF = low frequency, RMSSD = square root of the mean of the sum of the squares of differences between adjacent normal-to-normal (NN) intervals, SDNN = standard deviation of all NN intervals, VLF = very low frequency.
